# Anti-Inflammatory Activity of (Polyphenolic)-Sulfonates and Their Sodium Salts in Rodents

**DOI:** 10.1155/MBD.1998.67

**Published:** 1998

**Authors:** Iris H. Hall, Margaret E. Murphy, Amy L. Elkins

**Affiliations:** Division of Medicinal Chemistry and Natural Products School of Pharrnacy University of North Carolina Chapel Hill Chapel Hill North Carolina 27559-7360 USA

## Abstract

A series of polyphenolic-sulfonated compounds were observed to have potent anti-inflammatory activity and were protective against induced endotoxic shock in mice at 8 and 16 mg/kg, I.P. These agents proved to be potent elastase inhibitors in human leukocytes and J774-AI and IC-21 mouse macrophages as well as prostaglandin cyclo-oxygenase inhibitors in J774-AI macrophages. The compounds from 5 to 50 μM inhibited TNFα release from IC-21 macrophages and IL-1 release from mouse P388_D1_ macrophages induced by LPS. The binding of these cytokines to high affinity receptors on target cells, e.g. L929 fibroblasts and IL-2 in HuT78 T lymphoma cells, were also suppressed by the agents. These compounds blocked the adhesion of leukocytes and macrophages to the plasma membranes of L929 fibroblasts.


### ANTI-INFLAMMATORY ACTIVITY OF (POLYPHENOLIC)-SULFONATES AND THEIR SODIUM SALTS IN RODENTS

Iris H. Hall*, Margaret E. Murphy, and Amy L. Elkins

Division of Medicinal Chemistry and Natural Products, School of Pharrnacy, University of North Carolina Chapel Hill, North Carolina 27559-7360, USA

### ABSTRACT

A series of polyphenolic-sulfonated compounds were observed to have potent anti-inflammatory activity and were protective against induced endotoxic shock in mice at 8 and 16 mg/kg, I.P. These agents proved to be potent elastase inhibitors in human leukocytes and J774-AI and IC-21 mouse macrophages as well as prostaglandin cyclo-oxygenase inhibitors in J774-AI macrophages. The compounds from 5 to 50 μM inhibited TNFα release from IC-21 macrophages and IL-1 release from mouse $P388_{D1}$ macrophages induced by LPS. The binding of these cytokines to high affinity receptors on target cells, e.g. L929 fibroblasts and IL-2 in HuT78 T lymphoma cells, were also suppressed by the agents. These compounds blocked the adhesion of leukocytes and macrophages to the plasma membranes of L929 fibroblasts.

### INTRODUCTION

Cyclic imides[1-3], α-, β- and γ -alkylaminopropiophenones[4], amine-carboxyboranes and their metal complexes[5,6], thiosemicarbazones and their metal complexes [7], and triazolidinedione derivatives[8] have previously been shown to be potent anti-inflammatory and analgesic agents in rodents. These low molecular weight agents like other non-steroidal anti-inflammatory agents were able to block lysosomal hydrolytic and proteolytic enzyme activities as well as the synthesis of prostaglandins and leukotrienes, chemical mediators of inflammation. In addition they were able to protect against free radicals generated during inflammation. These small molecules blocked the release of cytokines synthesized by macrophages, i.e. TNFα and IL-1 and Il-6. The uniqueness of these agents is that they also competitively displace cytokines from binding to their high affinity receptors on L929 cells. Few reports in the literature indicate interaction at these receptor sites other than by peptides or antibodies. Suramin, a polysulfate naphthylurea, has been shown to be an IL-6 high affinity receptor antagonist[6]. Blocking these receptors is desirable because the entire inflammation cascade of events is triggered by the early release of TNFα and IL-1. The therapeutic use of small molecular weight antagonists is more desirable than the use of large molecular weight glycoproteins or growth hormones to block the inflammation or shock process.

### *Materials and methods*

The polyphenolic-sulfonated compounds found in Table 1 were provided as a gift by Genelabs, Inc.. Radioisotopes were obtained from New England Nuclear [Dupont, Boston, MA] and substrates and co-factors were obtained from Sigma Chemical Co. [St. Louis, MO]. Pentosan sulfate was used as a comparable standard in the assays and was obtained from Sigma Chemical Co.. Cell lines were obtained from American Type Culture Collection [Rockville, MD].

### *In Vivo Tests*

Anti-inflammatory screen in mice: Male $CF_1$ male mice weighing 28-32g obtained from Jackson Lab. [Bar Harbor, MA] were used to screen agents at 8 or 16 mg/kg intraperitoneal [I.P.] x 2 administered 3 hr and 30 min prior to administering the irritant, according to Winter's protocol[9,10]. Evaluation of the induced edema was made by injecting 2% carrageenan in 0.9% saline into the plantar region of the foot. The opposite foot injected with 0.9% isotonic saline was used as a base line. Indomethacin (8 or 10 mg/kg), pentoxifylline (50 mg/kg) and phenylbutazone (50 mg/kg) were used as standards for comparison of activity.

Table 1 **Structures of Polyphenolic-sulfonate Compounds**

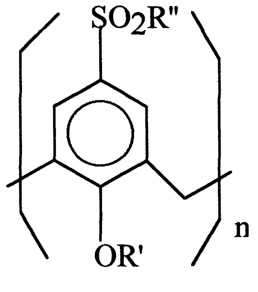

| Compound # | R' | R" | n |
|---|---|---|---|
| 1 | H | OH | 8 |
| 2 | H | OH | 6 |
| 3 | H | OH | 4 |
| 4 | $CH_3$ | OH | 8 |
| 5 | $CH_3$ | ONa | 4 |
| 6 | $CH_3$ | ONa | 6 |
| 7 | $OCH_3$ | ONa | 8 |
| 8 | OAc | ONa | 8 |

---

Protection against septic shock: $CF_1$ male mice (29-31g) were administered lipopolysaccharides [LPS] Salmonella abortus equi [Lot # 69F4003] [Sigma Chemical Co] at 10 mg/kg, I.P. which produced an $LD_{100}$ within 48-52 hr that was consistent with literature values[11]. Drugs from 2 to 16 mg/kg, I.P. were administered 2 hr prior and 2 hr post-injection of the LPS and then subsequently for every 24 hr for the length of the animals' lives. Deaths were recorded every 12 hr and continued for 96 hr. Indomethacin (8 mg/kg), dexamethasone (1 mg/kg) and pentoxyfylline (50 mg/kg) were used as standards.

*In vitro TNFα and IL-1 Measurements and Cellular Regulation*

IC-21 mouse macrophages were maintained in RPMI-1640 + 10% FCS + P/S. After the cells had grown to confluency, E. coli LPS at 10 μg/ml was added to the medium[12,13]. Agents were incubated at 12.5 to 100 μM final concentration for 18 hr. The medium (100 μl) was collected for TNFα determinations. Interleukin-1 [IL-1] release was determined using $P388_{D1}$ cells which were maintained in RPMI-1640 + 10% FCS + P/S. The L929 bioassay was used to quantitate the TNFα and the IL-1 levels[5,6]. The L929 mouse fibroblasts were grown in DMEM + 10% FCS + P/S to confluency in 96 well plates and incubated with 100 μl of medium from IC-20 or $P388_{D1}$ cells after 18 hr. The cells were stained with 0.2% crystal violet in 20% MeOH and read at 580 nm using a Molecular Devices scanner (SOFT-max program).

*High Affinity Binding to Receptors on L929-106 Cells*

Two μCi of $^{125}$I-TNFα (human recombinant, 30 mCi/μg New England Nuclear) or $^{125}$I-IL-1 (70-120μ Ci/μg,New England Nuclear) was added to confluency L929 cells for 90 min or 5 hr, respectively with 25, 50 and 100 μM of the test compounds [12]. The cells were washed repeatedly with isotonic saline, pH.7.2 and aliquots counted.

*Prostaglandin cylcooxygenase activity*

Mouse J774AI macrophages maintained in Dulbecco's modified Eagle's medium + 15% FCS + P/S. Cells (5 x $10^6$) were incubated with agents (5 to 100 μM final concentration) and $^3$H-arachidonic acid (100 Ci/mol) for 60 min at 37°C in a $CO_2$ incubator. The reaction was terminated with 2N HCl, the mixture extracted 2 X with ether, and the organic layer evaporated. The residue was dissolved in ethyl acetate and plated on TLC silica gel plates. These were eluted with chloroform:methanol:water:acetic acid [90:8:1:0.8] [13,14]. The plates were developed in iodine vapor and scraped according to the $R_f$ values of standard prostaglandins, and counted in a Packard scintillation beta counter correcting for quenching.

*Elastase Activity*

The substrate N-succinyl-L-alanyl-L-alanyl-L-alanine -*p*-nitroanilide was used in this assay and the hydrolytic product *p*-nitroanilide was determined at 410nm [15]. Porcine elastase EC 3.4.21.36 [Sigma Chemical Co.], human leukocytes or mouse macrophages were used as an enzyme source for the assay reaction incubated from 60 min to 30 hr. Drugs were incubated from 1 to 100 μM.

*Collagenase Activity*

Collagenase activity was determined using *Clostridium histolytricum* collagenase type I, 10 μg and N-[propionate 2,3-$^3$H]propionylated collagen incubated with agents present from 1 to 100 μM for 24 hr at 37°C [16]. The reaction was stopped with 1 ml 50 mM EDTA and the tube centrifuged at 10,000 *g* for 10 in. The supernatant was counted.

*Cell Adhesion*

RPMI 1788 leukocytes or J774 A1 mouse macrophages [$10^8$ cells] were incubated with $^3$H-thymidine [New England Nuclear, 78.3 Ci/mmole] for 3 hr in growth medium [17, 18]. The cells were centrifuged at 100 rpm for 5 min, washed in isotonic PBS, pH 7.2 and resuspended in fresh medium. Aliquots of labelled cells were added to confluent L929 mouse fibroblasts and drugs were added from 5 to 50 μM from 1-5 hr. The medium was decanted and L929 cells were washed repeatedly with PBS. The cells were taken up in 0.1 N NaOH and counted.

*Statistics*

In all of the tables, data is calculated as 100% of control $\pm$ the standard deviation. The statistical significance was determined by the Student's "t" test on the raw data.

### RESULTS

*<u>In Vivo</u> Pharmacology Tests*

The polyphenolic-sulfonate compounds proved to be potent anti-inflammatory agents and protected against endotoxin induced death in mice [Table 2 ]. Compounds **1-4** caused greater than 44% inhibition of induced edema at 8 mg/kg X 2 while compounds **1**, **4**, **6** and **8** afforded greater than 40% reduction of edema at 16 mg/kg X 2. Compound **8** was the most potent at 16 mg/kg X 2 with 62% reduction of edema. Indomethacin at 8 mg/kg afforded 26% reduction and at 10 mg/kg afforded 78% reduction of induced edema. Phenylbutazone at 50 mg/kg X 2 resulted in 47% reduction in edema. In the LPS induced endotoxin shock assay, the survival of control animals was 16% at 52 hr [Table 3]. Compounds **1, 2, 4** and **8** and pentosan sulfate resulted in 83% protection from death at 16 mg/kg. Compound **3** at 8 mg/kg and compounds **6** and **7** at 16 mg/kg resulted in 100% protection from LPS induced death. Compound **5** at 16 mg/kg resulted in 67% protection which was equal to dexamethasone at 1 mg/kg or pentoxifylline at 50 mg/kg.

*Cytokine release and binding to high affinity receptors*

TNFα release from IC-21 macrophages over an 18 hr period was significantly reduced by the agents **6**, **7**, and **8** following a concentration dependent effect from 5 to 50 μM. Compounds **7** and **8**, achieved greater than 50% reduction at 50 μM whereas compound **6** resulted in only 38% reduction [Table 4]. IL-1 release from P-$388_{D1}$ macrophages over 18 hr was also markedly reduced in a concentration dependent manner with >95% reduction at 25 and 50 μM concentration of compounds **6**, **7** and **8** [Table 5]. L929 TNFα high affinity receptor binding at 90 min., the optimum time for TNFα binding, was reduced by all three agents with greater than 50% suppression from 12.5 to 50 μM [Table 6]; drugs at the concentration of 25 μM afforded the best results with >75% reduction in TNFα binding. L929 fibroblast IL-1 high affinity receptor binding at 5 hr, the optimum time for IL-1 binding, was also reduced greater than 90% by the

Table 2 **Anti-inflammatory Activity of Polyphenolic-sulfonate Compounds I.P. in $CF_1$ Male Mice**

N =6 Percent of Control

| **Compound** | **8 mg/ Kg X 2** | **16 mg/Kg X 2** |
|---|---|---|
| Control | 100 + 5[a] | 100 + 5 |
| #1 | 51 + 4* | 56 + 5* |
| #2 | 47 + 4* | 74 + 5* |
| #3 | 58 + 6* | 61 + 5* |
| #4 | 56 + 6* | 48 + 4* |
| #5 | -- | 61 + 5* |
| #6 | -- | 48 + 3* |
| #7 | 56 + 5* | 72 + 5* |
| #8 | 70 + 6* | 38 + 3* |
| Pentosan polysulfate | -- | 49 + 4* |
| Indomethacin | 74 + 5* | 28 + 2* [10 mg/Kg X 2] |
| Pentoxifylline | -- | 70 + 6* [50 mg/Kg X 2] |
| Phenylbutazone | -- | [50 mg/Kg X 2] |

a = net increase of 84 mg edema

* $p \leq 0.001$

---

Table 3 **The Anti-Endotoxic Action of the Polyphenolic-sulfonate Compounds in $CF_1$ Male Mice, I.P.**

N = 6 Percent Survival at 52 hr

| Compound | **2 mg/kg** | **4 mg/Kg** | **8 mg/Kg** | **16 mg/Kg** |
|---|---|---|---|---|
| Control | 16 | 16 | 16 | 16 |
| #1 | 33 | 50 | 50 | |
| #2 | 83 | 50 | 50 | 83 |
| #3 | 67 | 50 | 100 | 50 |
| #4 | 50 | 33 | 67 | 83 |
| #5 | | | 33 | 67 |
| #6 | | | 50 | 100 |
| #7 | 67 | 67 | 50 | 100 |
| #8 | 67 | 67 | 67 | 83 |
| Pentosan polysulfate | | | 67 | 83 |
| Pentoxifylline | | -- | 67 + 6* [50 mg/Kg/day] | |

---

agents at 5 to 50 µM for compounds **6** and **8** [Table 7]. Compound **7** caused greater than 80% reduction in IL-1 binding only at 50 µM. IL-8 high affinity binding to chinese hamster ovarian carcinoma K-1 cells was reduced ~45% by compound **6** [Table 8] at 5 hr and 24 hr [Table 8]. Compounds **7**, **8** and pentosan sulfate at 50 µM caused only 21-23% reduction at 5 hr but were more effective at 24 hr µM causing > 40% reduction. IL-2 high affinity binding to HuT78 T lymphoma cell membrane receptors at 5 hr was reduced 45% at 50 µM of compounds **6, 8** and pentosan sulfate. These effects were not as evident at 2 hr but compound **8** at 50 µM caused 62% reduction of Il-2 binding and compound **7** resulted in a 31% reduction of Il-2 binding to its high affinity receptor [Table 9].

*Enzyme Assays*

Compounds **2** and **4** demonstrated potent inhibition of J774-A1 macrophage elastase activity at 60 min with $IC_{50}$ values of 0.79 and 0.03 X $10^{-5}$ M. Compounds **3** , **7** and **8** were effective with $IC_{50}$ values of 1-3 X $10^{-5}$ M and all other compounds were not active at these concentrations. Incubation for 120 min resulted in loss of elastase inhibition by the compounds with $IC_{50}$ values from 2.5 to 11 X $10^{-5}$ M at both 60 and 120 min. Human leukocyte elastase activity was inhibited similarly with $IC_{50}$ values from 4.5 -28 X $10^{-5}$ M [Table 10]. Porcine elastase was inhibited at 2, 8 and 30 hr by the agnets but the $IC_{50}$ values

Table 4 **TNFα Release from IC-21 Macrophages After 18 Hours Exposure to LPS**
N = 6 Percent of LPS Control

| **Concentration μM** | **LPS Salmonella 10 ug/ml** | **Compound #6** | **Compound #7** | **Compound #8** |
|---|---|---|---|---|
| 0 | 100+6[a] | | | |
| 5 | ------- | 118 + 7 | 113 + 5 | 73 + 4* |
| 12.5 | ------- | 102 + 5 | 88+ 6 | 66 + 4* |
| 25 | ------ | 86 + 6 | 84 + 5 | 44 + 3* |
| 50 | ------ | 62 + 4* | 47 + 4* | 53 +4* |

a = 150 pg/ml
* $p \leq 0.001$

Table 5 **Effects of Polyphenolic-sulfonates on TNFα Binding to L929 Fibroblasts High Affinity Receptors**
N = 6 Percent of Control

| **Concentration μM** | **Compound #6** | **Compound #7** | **Compound #8** |
|---|---|---|---|
| 0 | 100 + 5[a] | 100 + 5 | 100 + 5 |
| 5 | 61 + 5* | 18 + 3* | 59 + 6* |
| 12.5 | 39 + 4* | 8 + 2* | 43 + 4* |
| 25 | 24 + 3 | 20 + 3* | 23 + 4* |
| 50 | 36 + 5* | 17 + 3* | 37 + 4* |

a = 12819 cpm/mg
protein* $p \leq 0.001$

Table 6 **Effects of Polyphenolic-sulfonates on IL-1 Release from P-388$_{D1}$ Cells over 18 Hours**
N = 6 Percent of Control

| **Concentration μM** | **Compound #6** | **Compound #7** | **Compound #8** |
|---|---|---|---|
| 0 | 100 + 5[a] | 100 + 5 | 100 + 5 |
| 5 | 13 + 3* | 71 + 6* | 75+ 5* |
| 12.5 | 6 + 2* | 58 + 6* | 66 + 4* |
| 25 | 0 | 0 | 0 |
| 50 | 0 | 1 + 1* | 0 |

a = 186 ng/ml medium. * $p \leq 0.001$

Table 7 **Effects of Polyphenolic-sulfonates on $^{125}$I-IL-1 High Affinity Binding to L929 Fibroblasts at 8 Hours**
N = 6 Percent of Control

| **Concentration μM** | **Compound # 6** | **Compound # 7** | **Compound # 8** |
|---|---|---|---|
| 0 | 100 + 6[a] | 100 + 6 | 100 + 6 |
| 5 | 3 + 2* | 88 + 7 | 9 + 1* |
| 12.5 | 3 + 2* | 104 + 8 | 10 + 5* |
| 25 | 3 + 2* | 123 + 6* | 9 + 5* |
| 50 | 3 + 1* | 18 + 8* | 7 + 4* |

a = 16774 cpm/mg protein. * $p \leq 0.001$

were in the range of 5-9 X $10^{-4}$ M. *Clostridium histolytricum* collagenase type I activity was not inhibited significantly from $10^4$ to $10^6$ M concentration of drugs after 24 hr incubation. J774-A1 prostaglandin cyclooxygenase activity was suppressed by the agents with IC50 values of 2.06 to 7.62 X $10^{-5}$ M [Table 10].

Table 8 **Effects of Polyphenolic-sulfonates on $^{125}$I-IL-8 High Affinity Binding to Chines Hamster Ovarian Carcinoma Cell K-1**

N = 6 Percent of Control

| **Concentration μM** | **Compound # 6** | **Compound # 7** | **Compound # 8** | **Pentosan polysulfate** |
|---|---|---|---|---|
| 5 Hours | | | | |
| 0 | 100 + 5[a] | 100 + 5 | 100 + 5 | 100 + 5 |
| 50 | 54 + 5* | 77 + 5* | 79 + 4* | 79 + 6* |
| 24 Hours | 100 + 6[b] | 100 + 6 | 100 + 6 | 100 + 6 |
| 50 | 65 + 7* | 58 + 3* | 58 + 7* | 51 + 6* |
| 100 | 55 + 4* | 63 + 7* | 51 + 6* | 47 + 8* |

a = 190 cpm, b = 910 cpm. *p ≤ 0.001

---

Table 9 **Effects of Polyphenolic-sulfonates on $^{125}$I -Il-2 High Affinity Binding to HuT-8 Cells**

N = 6 Percent of Control

| **Concentration μM** | **Compound #6** | **Compound #7** | **Compound #8** | **Pentosan Polysulfate** |
|---|---|---|---|---|
| 2 Hours | 100 + 6[a] | 100 + 6 | 100 + 6 | 100 + 6 |
| 5 | 142 + 6* | 165 + 7* | 138 + 5* | 362 + 9* |
| 12.5 | 115 + 5 | 130 + 5* | 215 + 7* | 188 + 5* |
| 25 | 85 + 5 | 119 + 6 | 88 + 6 | 123 + 7 |
| 50 | 81 + 4* | 69 + 3* | 38 + 4* | 104 + 6 |
| 5 Hours | 100 + 7[b] | 100 + 7 | 100 +7 | 100 + 7 |
| 5 | 100 + 6 | 137 + 6* | 85 + 5 | 148 + 5* |
| 12.5 | 92 + 6 | 100 + 7 | 78 + 4 | 89 + 6 |
| 25 | 78 + 5* | 96 + 5 | 59 + 5* | 81 + 6 |
| 50 | 55 + 4* | 81 + 6 | 55 + 6* | 55 + 4* |

a = 2 hours -13 cpm; b = 5 hours -135 cpm/mg protein * p < 0.001

---

Table 10 **$IC_{50}$ Values as $10^{-5}$ M For Enzyme Inhibition of Polyphenolic Sulfates in Mouse Macrophages and Human Leukocytes**

| **Enzyme Activity** | **Elastase** | | | | | | **Cyclo-oxygenase** |
|---|---|---|---|---|---|---|---|
| | | **J774-A1** | **IC-21** | | **RPMI** | **1788** | **J744-A1** |
| Cmp'd # | 60 min | 120 min | 60 min | 120 min | 60 min | 120 min | 60 min |
| #1 | NA | 36 | 5.4 | 4.5 | 5.7 | 5.6 | 2.06 |
| #2 | 0.79 | 24 | 6.7 | 5.7 | 6.7 | 5.7 | 7.62 |
| #3 | 2.88 | 28 | 7.3 | 2.5 | 7.3 | 2.5 | 2.77 |
| #4 | 0.03 | 27 | 5.7 | 5.6 | 5.4 | 4.5 | 5.92 |
| #5 | NA | Increased | --- | --- | 9.0 | 11.1 | 6.51 |
| #6 | NA | Increased | --- | --- | 27.5 | 16.2 | NA |
| #7 | 1.64 | Increased | --- | --- | | | NA |
| #8 | 2.45 | 35 | 9.0 | 11.1 | | | 3.33 |
| Pentosan poly-sulfate | NA | Increased | | | | | NA |

NA = not active in enzyme assay

Table 11 **The Effects of Polyphenolic-sulfonates on the Adhesion of Human Leukocytes RPMI-1788 to Confluent L929 Fibroblasts Over Time**

N = 6 Percent of Control

| | | | | |
|---|---|---|---|---|
| 60 min | | | | |
| **Control** 100 + 6[a] | **Compound #6** | **Compound #7** | **Compound #8** | |
| 5 | 46 + 4* | 39 + 3* | 49 + 7* | |
| 12.5 | 49 + 6* | 35 + 5* | 40 + 5* | |
| 25 | 30 + 3* | 40 + 4* | 41 + 5* | |
| 50 | 41 + 5* | 49 + 4* | 55 + 4 | |
| 90 min | | | | |
| **Control** 100 + 7[b] | **Compound #6** | **Compound #7** | **Compound #8** | |
| 5 | 62 + 5* | 62 + 5* | 108 + 6 | |
| 12.5 | 55 + 6* | 55 + 5* | 65 + 6* | |
| 25 | 71 + 5* | 65 + 5* | 63 + 3* | |
| 2 Hours | | | | |
| **Control** 100+5[c] | **Compound #6** | **Compound #7** | **Compound #8** | **Pentosan polysulfate** |
| 12.5 | 125 + 5 | 108 + 7 | 92 + 6 | 83 + 5 |
| 25 | 100 + 6 | 101 + 6 | 108 + 4 | 116 + 6 |
| 50 | 108 + 6 | 104 + 8 | 116 + 6 | 147 + 6 |
| 5 Hours | | | | |
| **Control** 100+5[d] | **Compound #6** | **Compound #7** | **Pentosan polysulfate** | |
| 12.5 | 92 + 4 | 83 + 6 | 82 + 5 | |
| 25 | 95 + 5 | 92 + 5 | 82 + 6 | |
| 50 | 88+ 7 | 83 + 5 | 82 +.5 | |

a = 5025 cpm/mg protein; b = 2745 cpm/mg protein; c = 360 cpm/mg protein; d = 300 cpm/mg protein

* $p \leq 0.001$

---

Table 12 **The Effects of Polyphenolic sulfonates on the Adhesion of J-774 A1 Mouse Macrophages to Confluent L929 Fibroblasts**

N = 6 Percent of Control

| | | | | |
|---|---|---|---|---|
| 90 min. | | | | |
| **Control** 100+5[a] | **Compound #6** | **Compound #7** | **Compound #8** | **Pentosan polysulfate** |
| 5 | 137 + 6* | 302 + 8* | 136 + 5* | 329 + 12* |
| 12.5 | 133 + 5* | 196 + 6* | 135 + 7* | 363 + 16* |
| 25 | 128 + 7* | 155 + 7* | 183 + 7* | 260 + 9* |
| 50 | 103 + 7* | 153 + 8* | 94 + 6 | 176 + 6* |
| 2 Hours | | | | |
| **Control** 100+6[b] | **Compound #6** | **Compound #7** | **Compound #8** | **Pentosan polysulfate** |
| 5 | 123 + 6* | 123 + 5* | 73 + 5* | 75 + 7 |
| 12.5 | 77 + 6* | 85 + 6 | 61 + 6* | 53 + 6* |
| 25 | 118 + 7 | 68 + 6* | 91 + 5 | 66 + 5* |
| 50 | 122 + 6 | 117 + 7 | 92 + 6 | 110 + 6 |
| 5 Hours | | | | |
| **Control**100+ 8[c] | **Compound #6** | **Compound #7** | **Compound #8** | **Pentosan, polysulfate** |
| 5 | 88 + 5 | 84 + 6 | 107 + 5 | 103 + 5 |
| 12.5 | 109 + 6 | 121 + 6 | 106 + 6 | 108 + 7 |
| 25 | 131 + 6* | 118 + 6 | 125 + 7* | 169 + 6* |
| 50 | 119 + 5 | 105 + 4 | 131 + 7* | 187 + 7* |

a = 1380 cpm/ mg protein; b = 1490 cpm/mg protein; c = 2755 cpm/ mg protein . * $P \leq 0.001$

*Cell adhesion*

Compounds **6**, **7**, and **8** were effective from 5 to 50 μM in reducing significantly the adhesion of human leukocytes to confluent L929 fibroblasts at 60 and 90 min but were not effective at 2 and 5 hr [Table 11]. The agents were not as effective in reducing J774A1 macrophage adhesion to L929 fibroblasts at these concentrations [Table 12]. At 2 hr at 12.5 μM, they caused 15% to 39% reduction, yet pentosan polysulfate caused 47% reduction at 12.5 μM.

### DISCUSSION

A number of small molecular weight agents, e.g. amine-carboxyboranes and their metal complexes, indazolones, 3-imino-1-oxoisoindolines, triazolidinedione derivatives, α, β, and γ alkylaminoketones, and thiosemicarbazones as well as their metal complexes afforded similar reductions in induced edema as the polyphenolic sulfonates. These non-steroidal anti-inflammatory agents suppress both TNFα and IL-1 release and cytokine receptor binding from 25 to 100 μM similar to the current polyphenolic sulfonates. These agents as well as the polyphenolic sulfonates were very effective in protecting against LPS induced shock. Shock is mediated by the release of these regulatory cytokines, i.e TNFα and IL-1 and their binding to receptors on target inflammation cells which causes the release of inflammation enzymes and chemical mediators as well as enhancing adhesion of inflammatory cells to tissue lesions. It is difficult to determine whether the effects of the agents are direct or indirect in acting as antagonists of high affinity receptors for cytokine regulation, but they did effectively block elastase and cyclooxygenase activities and cell adhesion. The cytokines, e.g. TNFα, are large glycoproteins which exist as a trimer, folding so that only three small regions interact with specific high cysteine regions of the high affinity receptors. Mutagenic studies have indicated that only a few amino acid residues are important to binding of TNFα. The oligosaccharides of the cytokine molecule are thought to play a role in the binding of the cytokine to its receptor, since alterations of their structural composition leads to decreased binding of the cytokine molecule to the high affinity receptors. Sulfamoylthiophenone and sulfamoylpyrazole carboxylic acid derivatives have recently been shown to be anti-inflammatory and analgesic agents [19]. Thus, the polyphenolic sulfonates may play a similar role at these receptor binding sites, competing with the endogenous cytokines. Since TNFα regulates IL-1, IL-6 and IL-8 as well as its own release, there is a possible cross-over in the effects of agents on the cytokine receptors. More than one receptor exists on cells for some of the cytokines, i.e. TNFα and IL-1. What is most significant concerning these findings is that it may be possible to modulate cytokine effects on metabolism, proliferation, immunomodulation, etc., without using a large molecular weight protein that has deleterious side effects such as allergy and anaphaxisis. Numerous small molecular weight agents such as flavones, thalidomide, cyclosporin A, methotrexate, dexamethasone, or pentoxifylline, block TNFα or Il-1 synthesis or release from macrophages. Tenidap, which has structural features similar to the cyclic imides, appears to effect both Il-1 and TNFα production and activation from human monocytes. All of the anti-inflammatory and anti-rheumatoid agents found to date require in vivo doses 3-50 mg/kg, yet the studies in tissue culture to block cytokine synthesis and release require IC50 values in the 0.2 to 0.5 mM range, i.e. IC50 value for pentamidine is 1 mM, IX 207-887 is 30-60 mM and hydrocortisone is 10 mM. The drug RP 54-745 decreases mRNA synthesis of both TNFα and IL-1 after challenge with LPS. It is active in vivo at 25 mg/kg in mice but in vitro studies required 3 - 10 mM concentrations. Thus, the effect of polyphenolic sulfonate follows a similar pattern to other small molecular weight agents on cytokine modulation of the inflammation process. Because of these observations further investigation into their properties as potential therapeutic agents is needed.

### ACKNOWLEDGEMENTS

The authors wish to thank Dr. Tom Lee, Genelabs Incorporated, 505 Penobscot Dr., Redwood City CA, 94063 for the agents used in this project.

### REFERENCES

1. Tse E, Butner L, Huang Y, Hall IH. Arch. Pharm. [Med. Chem.] 1996; **329:** 35-40.
2. Butner L, Huang Y, Tse E, Hall IH. Biomed. Pharmacol. 1996; **50:** 290-296.

3. Hall IH, Simlot R, Oswald C, Murthy ARK, ElSourady H, Chapman J, Jr. Acta Pharm. Nord. 1990; **2:** 387-400.
4. Huang Y, and Hall I.H. Pharm. Toxicol. 1996; **1** : 17-38.
5. Hall III, Rajendran KG, Chen SY, Norwood VN, Morse KW, Sood A, Spielvogel, BF: Appl. Org.Metal. Chem. 1994; **8:** 473-480.
6.. Hall IH, Rajendran KG, Chen SY, Wong OT, Sood A, Spielvogel BF. Arch. Pharm. (Weinheim) 1995; **328:** 39-44.
7. Hall IH, Chen SY, Rajendran KG, West DX. Appl. Metal. Org. Chem. 1996; **10:** 485-493.
8. Hall IH, Simlot R, Izydore RA. Arch. Pharm. (Weinheim) 1995; **328:** 5-10.
9. Winter CA, Risley E, Nuss G. Proc. Soc. Exp. Bio. Med.1962; **111:** 544-547.
10. Roszkowski AP, Rooks WH,Tomonlonis A, Miller LM. J. Pharm. Exp. Ther. 1971; **179:** 114-123.
11. Noronha-Blob L, Lowe VC, Weitzberg M, Burch RM. Eur. J. Pharm. 1991; **199:** 387-388.
12. Kull FC, Jr. Nat. Immun. Cell Growth Regul. 1988; **7:** 254-265.
13. Tomlinson RV, Ringold HJ, Qureshi MC, Forchieli E. Biochem. Biophys. Res. Commun. 1971; **46:** 552-559.
14. Glatt M, Kalin H, Wagner K, Brune K. Agents Actions 1977; **7:** 321-326.
15. Kleinerman J, Ranga V, Rynbrant J, Sorensen J, Powers J. Am. Rev. Respir. Dis. 1980; **121:** 381-387.
16. Hu CL, Crombie G, Franzblau C. Anal. Biochem. 1978; **88:** 638-643.
17. Chin YH, Falanga V, Taylor JR, Cai JP. J. Invest. Dermatol. 1990; **94:** 413-417.
18. Murphy ME, Elkins AL, Shrewsbury RP, Sood A, Spielvogel BF, Hall IH. Metal Based-Drugs 1996; **3:** 31-47.
19. Diaz JA, Morante ME, Delgado L. Arch Pharm. Med. Chem.1996; **329:** 229-238.

**Received: January 6, 1998 - Accepted: January 14, 1998 - Received in revised camera-ready format: February 2, 1998**

